# Prevalence of ventilator-associated pneumonia and associated factors among intubated adult patients admitted in public hospitals in Addis Ababa, Ethiopia: a facility-based retrospective study design

**DOI:** 10.3389/fmed.2025.1500901

**Published:** 2025-04-25

**Authors:** Estibel Mengist Tegegne, Birhanu Chekol Gete, Dereje Bayissa Demissie

**Affiliations:** School of Nursing, St. Paul’s Hospital Millennium Medical College, Addis Ababa, Ethiopia

**Keywords:** intubation, prolonged, mechanical ventilation, prevalence, tracheostomy, ventilator-associated, pneumonia

## Abstract

**Background:**

Ventilator-associated pneumonia (VAP) occurs after 48 h of intubation or tracheostomy, leading to prolonged mechanical ventilation, increased healthcare costs, the emergence of antibiotic-resistant bacteria, and increased morbidity and mortality in resource-limited settings, including Ethiopia.

**Objectives:**

This study aimed to determine the prevalence of ventilator-associated pneumonia and identify associated factors among intubated adult patients admitted to public hospitals in Addis Ababa, Ethiopia, in 2024.

**Methods:**

A facility-based retrospective study was conducted on 341 adult patients admitted to the intubated Intensive Care Unit (ICU) from 1 January 2021 to 30 December 2023. Variables with *P*-values <0.05 in the bi-variable analysis were considered statistically significant.

**Results:**

A total of 335 patient charts were included in the study, with a response rate of 98.2%. More than half of the participants, 191 (57%), were male. The median age of patients was 40 years (IQR: 26–56 years). The study determined that the prevalence of ventilator-associated pneumonia (VAP) was 31.3% (95% CI: 26.3–36.4%). This study identified the following factors that increased the odds of ventilator-associated pneumonia: age of participants ≥60 years (AOR: 3.2, 95% CI: 1.51–7.12), re-intubation (AOR: 4.8, 95% CI: 2.4–9.4), duration of the patient on a mechanical ventilator (AOR: 3.2, 95% CI: 1.4–7.2), tracheostomy (AOR: 2.5, 95% CI: 1.2–5.2), and emergency intubation (AOR: 2.4, 95% CI: 1.3–4.6). These factors were significantly associated with VAP.

**Conclusion:**

This study determined that nearly one-third of study participants developed VAP and identified factors that increased the odds of VAP, including: advanced age (AOR: 3.2, 95% CI: 1.51–7.12), re-intubation (AOR: 4.8, 95% CI: 2.4–9.4), duration of the patient on a mechanical ventilator (AOR: 3.2, 95% CI: 1.4–7.2), tracheostomy (AOR: 2.5, 95% CI: 1.2–5.2), and emergency intubation (AOR: 2.4, 95% CI: 1.3–4.6). Policymakers and health planners should address these factors to improve patient outcomes and healthcare costs among intubated adult patients admitted to public hospitals in Addis Ababa.

## Introduction

Ventilator-associated pneumonia (VAP) is defined as pneumonia that develops more than 48 h after endotracheal intubation or tracheostomy, in the absence of any pneumonia symptoms at the time of intubation, admission, or tracheostomy (1, 2). Ventilator-associated pneumonia (VAP) is a new onset of pneumonia in ventilated patients, occurring between 48 h after mechanical ventilation and 48 h after extubation. It can be classified as early- or late-onset, with early-onset occurring within the first 4 days and late-onset occurring after 5 days (3).

A high level of clinical suspicion, along with a bedside examination, radiographic examination, and microbiologic investigation of respiratory secretions, are necessary to diagnose VAP (4). The 2016 clinical practice guidelines from the Infectious Diseases Society of America and the American Thoracic Society state that ventilator-associated pneumonia is diagnosed when a chest x-ray shows a new or changing lung infiltrate, along with at least two of the following clinical features: fever (≥38°c), increased white blood cell count (≥ 12*10^9^ WBC/ml), and purulent tracheobronchial secretions (1).

VAP was linked to a higher risk of hospital death and continues to be the most common infection among patients admitted to the intensive care unit (ICU). It is also associated with higher economic costs, longer lengths of stay attributable to the hospital, and higher mortality, particularly when lung infections are brought on by high-risk pathogens such as methicillin-resistant *Staphylococcus aureus* (MRSA), Gram-negative bacteria that produce extended-spectrum ß-lactamase (ESBL), multiple drug resistance (MDR) *Pseudomonas aeruginosa*, and *Acinetobacter baumannii*. Certain host, environmental, or pharmaceutical factors may increase a patient’s risk of developing VAP (5–8). VAP bundles of preventative actions need to be administered to patients who are at risk for VAP. The prevalence and burden of ventilator-associated pneumonia are significantly reduced when the ventilator-associated pneumonia bundle is followed (9).

Globally, the prevalence of ventilator-associated pneumonia is 15.6% (10). According to the 2016 clinical guidelines published by the American Thoracic Society (ATS) and the Infectious Diseases Society of America (IDSA), the death rate from VAP in the US could have reached up to 13% (11). A multicenter prospective study conducted in Europe found that the 30-day mortality rate of ventilator-associated pneumonia (VAP) was 29.9%, the early VAP mortality rate was 19.2%, and the late VAP mortality rate was 31.4% (12).

VAP patients spent an average of 8–24 days in the intensive care unit (ICU), while non-VAP patients spent 2.5–13 days there. Crude mortality rates for individuals with ventilator-associated pneumonia ranged from 16 to 94%, whereas those without the condition had a crude death rate of 0.2 to 51%. Additionally, the duration of ICU stay was significantly more prolonged in patients who developed late-onset VAP, with an average of 21.04 days compared to 10.82 days in early-onset VAP cases. The length of stay (LOS) in the intensive care unit (ICU) was 10 days longer for patients with ventilator-associated pneumonia (VAP), and they also had a higher death rate. Patients with ventilator-associated pneumonia may experience a variety of consequences, such as MDR organism infection, atelectasis, acute respiratory distress syndrome (ARDS), and severe sepsis/septic shock. These issues increase the likelihood of cost and death (13–15).

A deliberate investigation is necessary to lower the morbidity and death rate of ventilator-associated pneumonia, a significant nosocomial infection among intubated ICU patients. Good knowledge of VAP and its associated factors is an important way to decrease its consequences and mortality (16). Studies have been conducted to assess the knowledge of VAP prevention among critical care nurses in Ethiopia. These studies suggest that there is a need for training and education for critical care nurses to improve their knowledge of VAP prevention (17). However, limited studies conducted in Ethiopia show the prevalence of ventilator-associated pneumonia and its associated factors. This study is crucial to determine the extent and contributing factors of ventilator-associated pneumonia.

## Methods

### Study design, population, setting, and period

A facility-based retrospective cross-sectional study design was conducted among all admitted adult patients’ cards who were put on a mechanical ventilator in intensive care units of selected public hospitals in Addis Ababa from 1 January 2021 GC to 30 December 2023 GC at St. Paul’s Hospital Millennium Medical College, AaBET Hospital, Yekatit 12 Hospital Medical College, and Zewditu memorial Hospital from 15 May to 30 May 2024. All patients aged 15 years and older who had been intubated and on a mechanical ventilator for at least 48 h in intensive care units of selected public hospitals in Addis Ababa from 1 January 2021 to 30 December 2023 (18). Patients who had pneumonia before mechanical ventilation and those who had died within 48 h after starting mechanical ventilation were excluded from the study.

### Sample size and sampling procedure

The sample size of this study was determined by using a prevalence of 27.9% (19) from a study conducted at Bahir Dar University, and the target sample size was 341 patients, calculated by using a single population proportion formula: the minimum sample size calculated for first objective was 310 with the 10% non-response rate. The final sample size was 341.

To determine the required sample size for both specific objectives by calculating on open Epi info version 7.2 software using factors associated with ventilator-associated pneumonia among intubated adult patients admitted with the following assumptions: 95% confidence interval, 5% margin of error, and a power of 80% by taking study findings from Table 1.

**Table 1:** Sample size calculation for the 2nd objective 2024.

Variables	Power by %	OR/RR	Ratio of exp to unexpect	% of outcomes inunexposed	Sample size	Final sample size with 10% non-respose rate	References
Tracheostomy	80	OR 3.24 RR 2.59	1:1	11.36	176	194	(35)
Reintubation	80	OR 3.25 RR 2.41	1:1	15.56	144	159	(35)
Supine head position	80	OR 4.66 RR 3.44	1:1	9.67	110	121	(16)
Use of H_2_ blocker	80	OR 5.64 RR 4.16	1:1	7.7	98	108	(16)
Emergency intubation	80	OR 5.32 RR 2.08	1:1	36.05	60	66	(35)

Finally, the required sample size for this particular study was decided by taking the largest sample size, 341, which was included in the study.

#### Sampling procedure

Among public hospitals in Addis Ababa, four hospitals were selected using the lottery method. A simple random sampling technique was used, and the sampling frame was prepared by selecting patients on mechanical ventilator support using the patient registration book. Then, from the prepared sampling frame, the required number of samples was drawn using computer-generated methods.

**Figure 1:**
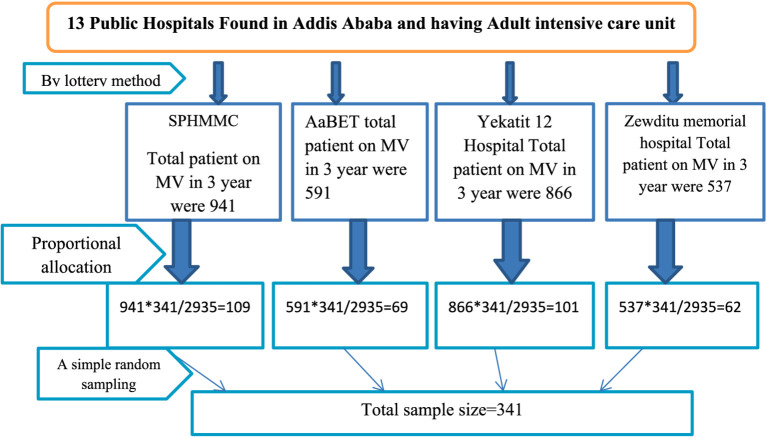
Schematic presentation of sampling procedure on ventilator-associated pneumonia and its associated factors among intubated adult patients admitted in public hospitals in Addis Ababa, Ethiopia, 2024.

The proportional allocation of patient charts in selected hospitals based on the number of patients on MV from 1 January 2021 to 30 December 2023 is detailed in Figure 1.

### Operational definitions

Ventilator-associated pneumonia (VAP): patients who were admitted to an adult intensive care unit and on a mechanical ventilator or tracheostomy and had a diagnosis of ventilator-associated pneumonia by the treating physician on their cards.

Early-onset VAP: Ventilator-associated pneumonia occurring within the first 4 days of mechanical ventilation.

Late-onset VAP: Ventilator-associated pneumonia occurring after 5 days of mechanical ventilation.

Low Glasgow Coma Scale (GCS): patients having less than nine GCS during admission to the ICU.

Incomplete chart: considered when 5% of the independent variable and/or the dependent variable’s indicator are not recorded.

### Data collection tools and techniques

Data were extracted through a data abstraction checklist from the patient’s registration book, and medical records of all patients who were intubated were selected. Then, the charts of the selected patients were reviewed in detail. The abstraction checklist was adapted from previously studied literature (13, 19–21). It contains sociodemographic characteristics, patient admission diagnosis conditions, and ICU intervention conditions. First, a letter of permission was sent to the ICU of each hospital, and the principal investigator communicated with the hospital administrator to briefly describe the study’s aim and obtain permission to conduct the study. Data were collected by two trained BSc Nurses under the supervision of one MSc nurse using KoboCollect software through chart review. Eligible patients were identified by data collectors.

### Data quality control

One day of training was given to the data collectors by the principal investigator on how to collect data, and the collected data were checked for completeness and accuracy on the same day of collection. A pretest was conducted on 5% of the sample size of intubated intensive care unit patient cards in each selected hospital. Based on the pretest findings, some modifications were made to the data extraction checklists. Reliability and internal consistency were measured using Cronbach’s alpha, which was 0.72.

### Data processing and analysis methods

The data were filled out by the data collectors using the KoboCollect software and were exported to SPSS version 26 for data processing and analysis. During the analysis and description of the study, variables were identified using frequency tables, proportions, percentages, graphs, and numerical summary measures. The chi-square assumption was checked before fitting the binary logistic regression. The association between each independent and dependent variable was assessed using binary logistic regression. Then, those independent variables with a *P*-value of <0.25 were transported to multivariate logistic regression to control the confounders. Hosmer–Lemeshow goodness-of-fit test was used to check the model fitness (0.797), and multicollinearity was assessed using the variance inflation factor (VIF = 1.26). A *P*-value of <0.05 in the multivariate analysis was used as a criterion for the statistically significant association. The strength of the association was measured using the odds ratio with corresponding 95% confidence intervals (CIs).

## Results

### Sociodemographic characteristics

From the total 341 samples, 335 patient charts were enrolled in the study with a response rate of 98.2%. The remaining six (1.8%) patient charts were excluded due to mechanical ventilation for less than 48 h. More than half of the participants, 191 (57%), were male. The age of the patients was found to be between 15 and 83 years, and approximately 271 (80.9%) of the participants’ ages were <60 years. The median age of patients was 40 years (inter-quartile range of 26–56 years) (Table 2).

**Table 2:** Sociodemographic factors of VAP among intubated adult patients admitted in public hospitals in Addis Ababa, Ethiopia, 2024 GC (*n* = 335).

Variable	Category	Ventilator-Associated Pneumonia (VAP)		
		Yes (%)	No (%)	Total (%)
Sex	Male	68 (64.8%)	123 (53.4%)	191 (57%)
	Female	37 (35.2%)	107 (46.6%)	144 (43%)
Age	<60	65 (61.9%)	206 (89.6%)	271 (80.9%)
	≥60	40 (38.1%)	24 (10.4%)	64 (19.1%)

### Prevalence of ventilator-associated pneumonia (VAP)

**Figure 2:**
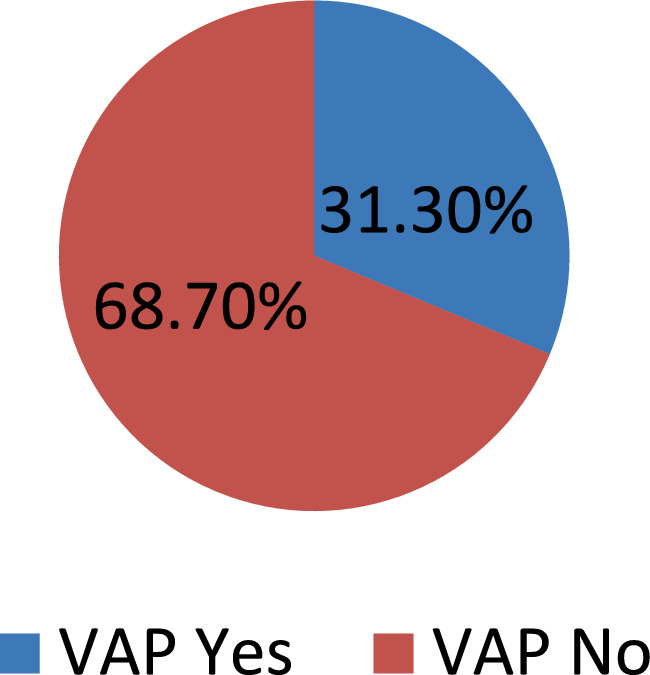
Prevalence of VAP among intubated adult patients admitted in public hospitals in Addis Ababa, Ethiopia, 2024 GC (*n* = 335).

The prevalence of Ventilator-Associated Pneumonia (VAP) was 31.3% (95% CI: 26.3–36.4%). Early-onset VAP occurred in 25 (23.8%), while late-onset VAP occurred in the remaining 80 (76.2%) patients (Figure 2).

### Intervention-related factors

The median lengths of patients on the mechanical ventilators were 15 days with an inter-quartile range of 8–21 days. The median lengths of stay of patients in the intensive care unit were 20 days and the inter-quartile range was 12–31 days. Approximately 113 (33.7%) of the participants have re-intubation; from these, 64.6% developed VAP, 207 (61.8%) participants used H2 blockers, and 98 (29.3%) of the participants have tracheostomy; from these, 64.2% developed VAP (Table 3).

**Table 3:** Intervention-related factors of VAP among intubated adult patients admitted in public hospitals in Addis Ababa, Ethiopia, 2024 GC (*n* = 335).

Variable	Category	Ventilator-Associated Pneumonia (VAP)		
		Yes (%)	No (%)	Total (%)
Re-intubation	Yes	73 (69.5%)	40 (17.4%)	113 (33.7%)
	no	32 (30.5%)	190 (82.6%)	222 (66.3%)
H2 blockers	Yes	57 (54.3%)	150 (65.2%)	207 (61.8%)
	No	48 (45.7%)	80 (34.8%)	128 (38.2%)
Tracheostomy	Yes	63 (60%)	35 (15.2%)	98 (29.3%)
	No	42 (40%)	195 (84.8%)	237 (70.7%)
Emergency intubation	Yes	57 (54.3%)	70 (30.4%)	127 (37.9%)
	No	48 (45.7%)	160 (69.6%)	208 (62.1%)
Supine head position	Yes	17 (16.2%)	45 (19.6%)	62 (18.5%)
	No	88 (83.8%)	185 (80.4%)	273 (81.5%)
Corticosteroid use	Yes	77 (73.3%)	178 (77.4%)	255 (76.1%)
	No	28 (26.7%)	52 (22.6%)	80 (23.9%)
Taken ≥ 3 antibiotics	Yes	39 (37.1%)	83 (36%)	122 (36.4%)
	No	66 (62.9%)	147 (64%)	213 (63.6%)
Blood transfusion	Yes	41 (39%)	91 (39.6%)	132 (39.4%)
	No	64 (61%)	139 (60.4%)	203 (60.6%)

### Admission diagnosis-related factors

**Figure 3:**
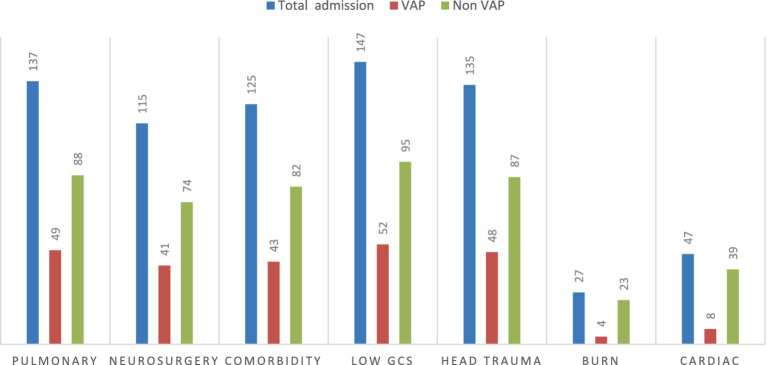
VAP status with common underlying comorbidity diseases among intubated adult patients admitted in public hospitals in Addis Ababa, Ethiopia, 2024 GC (*n* = 335). GCS, Glasgow Coma Scale.

Out of the total patient charts reviewed, 137 (40.9%) were admitted with a diagnosis of pulmonary disease, of which 49 (35.6%) developed VAP. Additionally, 135 (40.3%) were admitted with head trauma, and 48 (35.5%) of these patients developed VAP. In contrast, only 4 (3.8%) patients were admitted with burn injuries (Figure 3).

### Patient outcome

**Figure 4:**
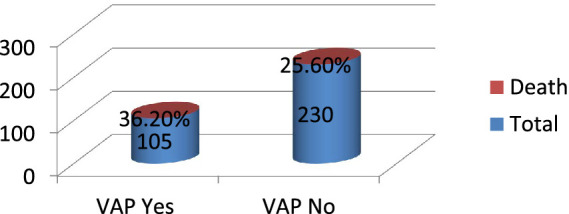
Patient outcome among intubated adult patients admitted in public hospitals in Addis Ababa, Ethiopia, 2024 GC (*n* = 335).

This study found that out of the 335 admitted patients sampled, 97 (29%) died. Among the 105 patients who developed VAP, 38 (36.2%) died, while among the 230 patients who did not develop VAP, 59 (25.6%) died (Figure 4).

### Bi-variable logistic regression analysis

The bi-variable logistic regression analysis identified 12 candidate factors for the multivariable logistic regression model. To be liberal, a *P*-value of 0.25 as a cutoff value was used to enter into multivariable logistic regression analysis; see details in Table 4.

**Table 4:** Bi-variable logistic regression analysis to identify factors of VAP among adult intensive care unit admitted patients in public hospitals in Addis Ababa, Ethiopia, 2024.

Variable	Category	Ventilator-Associated Pneumonia (VAP)				
		Yes	No	COR	95%CI	*P*-value
Age	<60	65 (61.9%)	206 (89.6%)	1	1	
	> = 60	40 (38.1%)	24 (10.4%)	5.282	2.964–9.413	0.000
Sex	Male	68 (64.8%)	123 (53.4%)	1.599	0.992–2.576	0.054
	Female	37 (35.2%)	107 (46.6%)			
Re-intubation	Yes	73 (69.5%)	40 (17.4%)	10.836	6.330–18.548	0.000
	No	32 (30.5%)	190 (82.6%)			
H2 blocker	Yes	57 (54.3%)	150 (65.2%)	0.633	0.396–1.013	0.057
	No	48 (45.7%)	80 (34.8%)			
Corticosteroid use	Yes	77 (73.3%)	178 (77.4%)	0.803	0.472–1.367	0.419
	No	28 (26.7%)	52 (22.6%)			
Tracheostomy	Yes	63 (60%)	35 (15.2%)	8.357	4.914–14.212	0.000
	No	42 (40%)	195 (84.8%)			
Day on MV	>14	90 (85.7%)	82 (35.7%)	10.829	5.887–19.922	0.000
	<=14	15 (14.3%)	148 (64.3%)			
Emergency intubation	Yes	57 (54.3%)	70 (30.4%)	2.714	1.687–4.368	0.000
	No	48 (45.7%)	160 (69.6%)			
Taken ≥3 antibiotics	Yes	39 (37.1%)	83 (36%)	1.047	0.648–1.689	0.852
	No	66 (62.9%)	147 (64%)			
Blood transfusion	Yes	41 (39%)	91 (39.6%)	0.979	0.610–1.570	0.928
	No	64 (61%)	139 (60.4%)			
Supine head position	Yes	17 (16.2%)	45 (19.6%)	0.794	0.430–1.466	0.461
	No	88 (83.8%)	185 (80.4%)			
Comorbidity	Yes	43 (41%)	82 (35.7%)	1.252	0.780–2.010	0.353
	No	62 (59%)	148 (64.3%)			
Pulmonary	Yes	49 (46.7%)	88 (38.3%)	1.412	0.885–2.251	0.147
	No	56 (53.3%)	142 (61.7%)			
Low GCS	Yes	52 (49.5%)	95 (41.3%)	1.394	0.877–2.217	0.160
	No	53 (50.5%)	135 (58.7%)			
Head trauma	Yes	48 (45.7%)	87 (37.8%)	1.384	0.867–2.209	0.173
	No	57 (54.3%)	143 (62.2%)			
Burn	Yes	4 (3.8%)	23 (10%)	0.356	0.120–1.058	0.063
	No	101 (96.2%)	207 (90%)			
Cardiac	Yes	8 (7.6%)	39 (17%)	0.404	0.182–0.898	0.026
	No	97 (92.4%)	191 (83%)			

*PV < 0.05 statistically significant, 1 = reference.

AOR, adjusted odds ratio; CI, confidence interval; COR, crude odds ratio; MV, mechanical Ventilator; GCS, Glasgow Coma Scale.

### Multi-variable logistic regression analysis

The bi-variable logistic regression analysis identified 12 candidate factors for the multivariable logistic regression model. To be liberal, a *P*-value of 0.25 as a cutoff value was used to enter into multivariable logistic regression analysis. Finally, five variables, including age, re-intubation, duration of the patient on a mechanical ventilator, tracheostomy, and emergency intubation, have had a significant association with VAP on multivariate binary logistic regression.

Patients whose ages were ≥60 years were 3.2 times more likely to develop VAP (AOR: 3.2, 95% CI: 1.51–7.12). Patients who had re-intubation were 4.8 times at higher risk of developing VAP (AOR: 4.8, 95% CI: 2.4–9.4). Patients who were on MV support for more than 14 days had a 3.2 times higher chance of developing VAP (AOR: 3.2, 95% CI: 1.4–7.2). Patients who had tracheostomy were 2.5 times more likely to develop VAP (AOR: 2.5, 95% CI: 1.2–5.2) than those who had not had tracheostomy. Patients who had emergency intubation had a 2.4 times higher chance of developing VAP (AOR: 2.4, 95% CI: 1.3–4.6) than those not undergoing emergency intubation (Table 5).

**Table 5:** Multivariable analysis to identify factors of VAP among adult intensive care unit admitted patients in public hospitals in Addis Ababa, Ethiopia, 2024.

Variable	Category	Ventilator-Associated Pneumonia (VAP)							
		Yes	No	COR	95%CI	*P*-value	AOR	95%CI	*P*-value
Age	<60	65 (61.9%)	206 (89.6%)	1			1		
	≥60	40 (38.1%)	24 (10.4%)	5.282	2.964–9.413	0.000	3.293	1.521–7.127	0.002 *
Sex	Male	68 (64.8%)	123 (53.4%)	1.599	0.992–2.576	0.054	0.946	0.496–1.802	0.865
	Female	37 (35.2%)	107 (46.6%)				1		
Re-intubation	Yes	73 (69.5%)	40 (17.4%)	10.836	6.330–18.548	0.000	4.808	2.439–9.478	0.000 *
	No	32 (30.5%)	190 (82.6%)				1		
H2 blocker	Yes	57 (54.3%)	150 (65.2%)	0.633	0.396–1.013	0.057	0.653	0.338–1.263	0.205
	No	48 (45.7%)	80 (34.8%)				1		
Tracheostomy	Yes	63 (60%)	35 (15.2%)	8.357	4.914–14.212	0.000	2.510	1.208–5.213	0.014 *
	No	42 (40%)	195 (84.8%)				1		
Day on MV	>14	90 (85.7%)	82 (35.7%)	10.829	5.887–19.922	0.000	3.242	1.445–7.276	0.004 *
	<=14	15 (14.3%)	148 (64.3%)				1		
Emergency intubation	Yes	57 (54.3%)	70 (30.4%)	2.714	1.687–4.368	0.000	2.452	1.305–4.606	0.005 *
	No	48 (45.7%)	160 (69.6%)				1		
Pulmonary	Yes	49 (46.7%)	88 (38.3%)	1.412	0.885–2.251	0.147	2.067	0.567–7.537	0.271
	No	56 (53.3%)	142 (61.7%)				1		
Low GCS	Yes	52 (49.5%)	95 (41.3%)	1.394	0.877–2.217	0.160	0.963	0.467–1.984	0.918
	No	53 (50.5%)	135 (58.7%)				1		
Head trauma	Yes	48 (45.7%)	87 (37.8%)	1.384	0.867–2.209	0.173	3.968	0.934–16.860	0.062
	No	57 (54.3%)	143 (62.2%)				1		
Burn	Yes	4 (3.8%)	23 (10%)	0.356	0.120–1.058	0.063	0.913	0.146–5.713	0.922
	No	101 (96.2%)	207 (90%)				1		
Cardiac	Yes	8 (7.6%)	39 (17%)	0.404	0.182–0.898	0.026	0.766	0.184–3.194	0.715
	No	97 (92.4%)	191 (83%)				1		

*Indicates statistically significant, 1 = reference.

AOR, adjusted odds ratio; CI, confidence interval; COR, crude odds ratio; MV, mechanical Ventilator; GCS, Glasgow Coma Scale.

## Discussion

This study aimed to determine the prevalence of VAP and identify factors of VAP among intubated adult intensive care unit admitted patients in public hospitals in Addis Ababa, Ethiopia. Despite the mechanical ventilator being an essential feature of modern intensive care unit service, it is associated with a substantial risk of VAP. In this study, 31.3% (95% CI: 26.3–36.4%) of patients developed VAP during their intensive care unit stay, with identified factors increasing the odds of VAP being advanced age, re-intubation, duration of the patient on a mechanical ventilator, tracheostomy, and emergency intubation, respectively.

This study found that the prevalence of VAP was 31.3% (95% CI: 26.3–36.4%). It is comparable with studies conducted in Bahir Dar (27.9%), Brazil (26.2%), Saudi Arabia (35.4%), and India (35%) (19, 22–24). However, this is lower than a study conducted in Egypt, which was 57.5% (25). This may be due to sociodemographic variation and the small sample size in the Egyptian study. In studies conducted in Iran, Tehran, and Turkey, the prevalence of VAP was 11, 21.6, and 15.4%, respectively (13, 20, 26), which was lower than this study’s finding. This difference may be due to differences in health facility setups and VAP diagnosis criteria.

In this study, patients aged ≥60 years were 3.2 times more likely to develop VAP (AOR: 3.2, 95% CI: 1.51–7.12) than patients aged less than 60 years, which is similar to other study findings such as studies conducted in Canada, China, and Kunming, China (27–29). The reason may be the decline of the physiological function of respiration, the gradual atrophy of respiratory muscles, the gradual reduction of lung tissue elasticity, the visibly weakened protective cough reflex, and the decreased immune function in the elderly (21).

In the current study, patients who were re-intubated were 4.8 times at higher risk of developing VAP (AOR: 4.8, 95% CI: 2.4–9.4) than patients who were not re-intubated, which is similar to the findings in studies conducted in Australia (30), Egypt, Pondicherry, and India (25, 31).

The possible justification might be an increased risk of aspiration of colonized oropharyngeal secretions into the lower airways in patients with subglottic dysfunction or impaired consciousness after several days of intubation and direct aspiration of gastric contents into the lower airways, particularly when a nasogastric tube is kept in place after extubation (32).

In this study, patients who were on mechanical ventilator support for more than 14 days were 3.2 times higher chance of developing VAP (AOR: 3.2, 95% CI: 1.4–7.2) than patients on MV support less than or equal to 14 days, which is similar to the findings in the studies conducted in Bahir Dar, Brazilian University Hospital, Egypt, Canada (19, 25, 27, 33). The possible justification for this may be that the artificial airway established by mechanical ventilation changes the mucosal defense function of the normal airway, and long-term ventilation increases the risk of infection, which is caused by humidifiers and ventilator loops that are the source of the pathogen due to exposure (30).

The current study found that patients who had tracheostomy were 2.5 times more likely to develop VAP (AOR: 2.5, 95% CI: 1.2–5.2) than those who had not had tracheostomy, which is similar to the findings in studies conducted in a tertiary care hospital in India, China, northern India, and southern Poland (28, 34–36). The possible justification may be increased tracheal colonization around the tracheostomy tube into the trachea because of leakage of pooled secretions, which leads to VAP. However, a study conducted at the University of Texas showed that early tracheostomy is independently associated with a lower rate of VAP. Performing early tracheostomy is associated with less chance of VAP (37). A possible justification maybe that it reduces the duration of mechanical ventilation and ICU stay of the patient compared to the patient with late tracheostomy.

In this study, patients who had emergency intubation had a 2.4 times higher chance of developing VAP (AOR: 2.4, 95% CI: 1.3–4.6) than those not undergoing emergency intubation, which is similar to the findings in studies conducted in a tertiary care hospital in India and northern India (34, 35). The possible justification may be that in emergency intubation, there is a breach of sterility, and the patient is at high risk of aspiration of secretions and GI contents.

However, in this study, there was no significant association between low GCS and VAP. This study finding is contradicted by a study conducted in Bahir Dar (19). The difference might be due to differences in the data analysis methods; the log-binomial analysis method was used in the studies in Bahir Dar, while logistic analysis was executed in this study.

### Limitations of the study

The retrospective cross-sectional study does not show a causal relationship, and selection bias is not avoidable. As secondary data, there may be data incompleteness.

## Conclusion

This study determined that nearly one-third of study participants developed VAP, and identified factors that increased the odds of VAP were advanced age (AOR: 3.2, 95% CI: 1.51–7.12), re-intubation (AOR: 4.8, 95% CI: 2.4–9.4), duration of the patient on a mechanical ventilator (AOR: 3.2, 95% CI: 1.4–7.2), tracheostomy (AOR: 2.5, 95% CI: 1.2–5.2), and emergency intubation (AOR: 2.4, 95% CI: 1.3–4.6). Addressing these factors through targeted preventive strategies such as precaution during emergency intubation, minimizing the occurrence of re-intubation, and optimizing ventilator weaning protocols could help reduce the substantial burden of VAP, enhance clinical practices, and improve patient outcomes and healthcare costs.

### Recommendations

Hospital administrative and Health care professionals should give great special attention to elderly patients, reduce patient length of stay on mechanical ventilation, strengthen precautions during emergency intubation, minimize the occurrence of re-intubation, and avoid tracheostomy as far as possible in a similar public hospital in resource-limited areas.

Policymakers and health planners should address these identified factors through targeted preventive strategies such as strengthening VAP bundle implementation and optimizing ventilator weaning protocols, which could help enhance clinical practices and improve patient outcomes and healthcare costs.

Further research is warranted to explore additional risk factors and potential interventions for VAP prevention.

## Data Availability

The raw data supporting the conclusions of this article will be made available by the authors, without undue reservation.
